# Percutaneous endoscopic necrosectomy with the assistance of implanted stent to manage walled-off necrosis: first clinical experience

**DOI:** 10.1055/a-2248-0376

**Published:** 2024-02-15

**Authors:** De-Liang Li, Hongmei Zhang, Jinglong Lv, Libo Quan, Dan Liu, Lixia Zhao, Bingrong Liu

**Affiliations:** 1 Department of Gastroenterology and Hepatology, The First Affiliated Hospital of Zhengzhou University, Zhengzhou, China; 2 State Key Laboratory of Esophageal Cancer Prevention and Treatment, The First Affiliated Hospital of Zhengzhou University, Zhengzhou, China

We report the case of a 46-year-old man with walled-off necrosis (WON) due to severe acute pancreatitis and failure of multiple and prolonged percutaneous catheter drainage procedures. As the cavity of WON was far from the gastrointestinal tract, we performed percutaneous endoscopic necrosectomy (PEN) through the sinus ( Video 1 ).

Percutaneous endoscopic necrosectomy with the assistance of an implanted stent.Video 1

Angiography showed the lesion of WON after injection of iohexol through the catheter placed beforehand ( Fig. 1 **a** ). A guidewire (Boston Scientific, Marlborough, Massachusetts, USA) was introduced through the drainage catheter with the guidance of radiation and coiled within the cavity. The preplaced drainage catheter was then removed. The sinus was dilated using a dilating bougie (Micro-Tech [Nanjing] Co., Jiangsu, China) with a diameter of 7–9–11–14 mm successively ( Fig. 1 **b** ). A lumen-apposing metal stent (LAMS), 22 mm in diameter and 80 mm in length (Micro-Tech [Nanjing] Co.) was delivered to the cavity through the sinus after full expansion. Necrosis was seen in the cavity under conventional therapeutic endoscopy ( Fig. 1 **c** ). A snare (Boston Scientific) was used to remove necrotic tissue ( Fig. 1 **d** ). The proximal flange of the stent was released outside the abdomen and the stent remained in place ( Fig. 1 **e** ). An ostomy bag was used to drain pus and necrotic debris from inside the cavity.

**Fig. 1:**
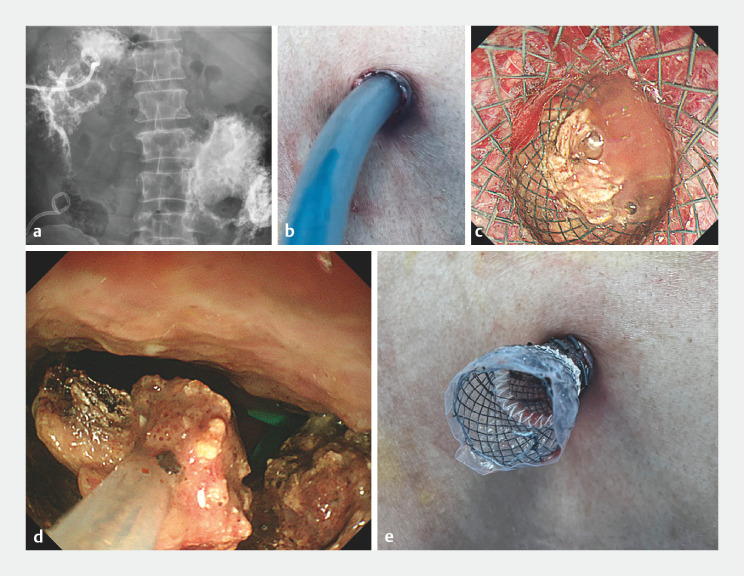
Percutaneous endoscopic necrosectomy with stent assistance for management of walled-off necrosis (WON). **a** Angiography showed the lesion of WON. **b** A dilating bougie was inserted into the lesion to dilate the sinus. **c** A lumen-apposing metal stent was delivered to the cavity of the WON; necrosis was present in the cavity. **d** A snare was used to remove necrotic tissue. **e** The proximal flange of the stent was released outside the abdomen and the stent remained in place for drainage and further percutaneous endoscopic necrosectomy procedures.

Two further PEN procedures were performed via the stent according to the patient’s condition. The stent was removed after the lesion subsided, and the opening of the sinus was sewn up.

PEN without stent assistance has been reported sporadically for the treatment of WON 1 2 3 . This procedure involves repeated expansion, and the need for nephroscopy or ultrathin endoscopy makes it a demanding operation. We placed LAMS in the sinus and kept the stent in situ temporarily, which was convenient for drainage and repeat PEN procedures. We believe that PEN with the assistance of stent placement is accessible, effective, and safe for the management of lateral refractory WONs.

Endoscopy_UCTN_Code_TTT_1AR_2AI

## Notes

Endoscopy E-Videos https://eref.thieme.de/e-videos
*E-Videos* is an open access online section of the journal *Endoscopy* , reporting on interesting cases and new techniques in gastroenterological endoscopy. All papers include a high-quality video and are published with a Creative Commons CC-BY license. Endoscopy E-Videos qualify for HINARI discounts and waivers and eligibility is automatically checked during the submission process. We grant 100% waivers to articles whose corresponding authors are based in Group A countries and 50% waivers to those who are based in Group B countries as classified by Research4Life (see: https://www.research4life.org/access/eligibility/ ). This section has its own submission website at https://mc.manuscriptcentral.com/e-videos .
